# Differential Gene Expression to Investigate the Effects of Low-level Electrochemical Currents on *Bacillus subtilis*

**DOI:** 10.1186/2191-0855-1-39

**Published:** 2011-11-11

**Authors:** Robert Szkotak, Tagbo H R Niepa, Nikhil Jawrani, Jeremy L Gilbert, Marcus B Jones, Dacheng Ren

**Affiliations:** 1 Department of Biomedical and Chemical Engineering, Syracuse University, Syracuse, NY 13244, USA; 2 Syracuse Biomaterials Institute, Syracuse University, Syracuse, NY 13244, USA; 3 J. Craig Venter Institute, Rockville, MD 20850, USA; 4 Department of Biology, Syracuse University, Syracuse, NY 13244, USA; 5 Department of Civil and Environmental Engineering, Syracuse University, Syracuse, NY 13244, USA

**Keywords:** *Bacillus subtilis*, bioelectric effect, biofilm, gene expression, electrochemical current

## Abstract

With the emergence and spread of multidrug resistant bacteria, effective methods to eliminate both planktonic bacteria and those embedded in surface-attached biofilms are needed. Electric currents at μA-mA/cm^2 ^range are known to reduce the viability of bacteria. However, the mechanism of such effects is still not well understood. In this study, *Bacillus subtilis *was used as the model Gram-positive species to systematically investigate the effects of electrochemical currents on bacteria including the morphology, viability, and gene expression of planktonic cells, and viability of biofilm cells. The data suggest that weak electrochemical currents can effectively eliminate *B. subtilis *both as planktonic cells and in biofilms. DNA microarray results indicate that the genes associated with oxidative stress response, nutrient starvation, and membrane functions were induced by electrochemical currents. These findings suggest that ions and oxidative species generated by electrochemical reactions might be important for the killing effects of these currents.

## Introduction

The rapid development and spread of multidrug resistant infections present an increasing challenge to public health and disease therapy (Alekshun and Levy 2003). As an intrinsic mechanism of drug resistance, biofilm formation renders bacteria up to 1000 times less susceptible to antibiotics than their planktonic (free-swimming) counterparts of the same genotype (Costerton et al. 1994). Such intrinsic mechanisms also facilitate the development of resistance through acquired mechanisms that are based on genetic mutations or drug resistance genes. Consistently, excessive antibiotic treatment of biofilm infections at sublethal concentrations has been shown to generate antibiotic-tolerant strains (Narisawa et al. 2008). Biofilms are responsible for at least 65% of human bacterial infections (Costerton et al. 2003). For example, it is estimated that in the United States 25% of urinary catheters become infected with a biofilm within one week of a hospital stay, with a cumulative 5% chance each subsequent day (Maki and Tambyah 2001). Biofilms are also detected on implanted devices and are a major cause of implant surgical removal (Hetrick and Schoenfisch 2006; Norowski and Bumgardner 2009). Orthopedic implants showed a 4.3% infection rate, or approximately 112,000 infections per year in the U.S. (Hetrick and Schoenfisch 2006). This rate increases to 7.4% for cardiovascular implants (Hetrick and Schoenfisch 2006), and anywhere from 5%-11% for dental implants (Norowski and Bumgardner 2009).

In the biofilm state, bacteria undergo significant changes in gene expression leading to phenotypic changes that serve to enhance their ability to survive in challenging environments. Although not completely understood, the tolerance to antibiotic treatments is thought to arise from a combination of limited antibiotic diffusion through the extracellular polymeric substances (EPS), decreased growth rate of biofilm cells, and increased expression of antibiotic tolerance genes in biofilm cells (Costerton et al. 1999). Common treatments that are capable of removing biofilms from a surface are by necessity harsh and often unsuitable for use due to medical or environmental concerns. It is evident that alternative methods of treating bacterial infections, and most notably biofilms, are required.

Electric currents/voltages are known to affect bacterial cells. However, most of the studies have been focused on high voltages and current levels such as eletctroporation, electrophoresis, iontophoresis, and electrofusion (Berger et al. 1976; Costerton et al. 1994; Davis et al. 1991; Davis et al. 1992) except for a few studies about biofilm control using weak electric currents. In 1992, Blenkinsopp et al. (1992) reported an interesting synergistic effect between 2.1 mA/cm^2 ^direct currents (DCs) and biocides in killing *Pseudomonas aeruginosa *biofilm cells. This phenomenon was named the "bioelectric effect" (Blenkinsopp et al. 1992; Costerton et al. 1994). In addition to *P. aeruginosa*, bioelectric effects have also been reported for *Klebsiella pneumoniae *(Stoodley et al. 1997; Wellman et al. 1996), *Escherichia coli *(Caubet et al. 2004), *Staphylococcus aureus *(del Pozo et al. 2009; Giladi et al. 2008), *P. fluorescens *(Stoodley et al. 1997), as well as mixed species biofilms (Shirtliff et al. 2005; Wellman et al. 1996). Although the impact of electric currents on bacterial susceptibility to antibiotics and biocides is well accepted, there is little understanding about the mechanism of bioelectric effect. An electric current at an electrode surface can trigger ion flux in the solution as well as electrochemical reactions of the electrode materials and redox species with electrolyte and generate many different chemical species, e.g. metal ions, H^+ ^and OH^-^. Although pH change has been shown to cause contraction of the biofilm formed on the cathodic electrode (Stoodley et al. 1997), change of medium pH to which prevails during electrolysis did not enhance the activity of antibiotics (Stewart et al. 1999). Consistent with this observation, buffering the pH of the medium during electrolysis failed to eliminate the bioelectric effect (Stewart et al. 1999). Another finding suggesting the existence of other factors is that the bioelectric effect has been observed for biofilms formed in the middle of an electric field, but not in contact with either the working electrode or counter electrode (Costerton et al. 1994; Jass et al. 1995). Since the electrochemically-generated ions accumulate around the electrodes, the biofilms in the middle of an electric field are not experiencing significant changes in pH or other products of electrochemical reactions. This is also evidenced by the report (Caubet et al. 2004) that radio frequency alternating electric current can enhance antibiotic efficacy. Since no electrochemically generated molecules or ions will likely accumulate with alternating currents, other factors may play a critical role. The bioelectric effect was also observed when the growth medium only contained glucose and two phosphate compounds. This observation eliminates the electrochemical reaction of salts as an indispensable factor of bioelectric effect (McLeod et al. 1999). Previous studies have also ruled out the impact of temperature change during electrolysis (less than 0.2°C) (Stewart et al. 1999). Although these studies provided useful information about bioelectric effect, its mechanism is still unknown. The exact factors causing bioelectric effect and their roles in this phenomenon remain elusive. Compared to biofilms, even less is known about the effects of weak electric currents on planktonic cells.

Many aspects of cellular functions are electrochemical in nature; e.g., the redox state of cells is related to membrane status, oxidative status, energy generation and utilization and other factors. Therefore, it is possible that the redox state of cells may be affected by electrochemical currents (henceforth ECs). To better understand the mechanism of bacterial control by ECs, we conducted a systematic study of the effects of weak ECs on the planktonic and biofilm cells of the model Gram-positive bacterium *Bacillus subtilis*. We chose *B. subtilis *because it is a typically used model Gram-positive organism in research (Zeigler et al. 2008) and allows us to compare with the data in our previous studies of its biofilm formation (Ren et al. 2004a; Ren et al. 2004b; Ren et al. 2002). It is important to control Gram-positive bacteria since they are responsible for 50% of infections in the United States, and 60% of overall nosocomial infections (Lappin and Ferguson 2009; Rice 2006). To the best of our knowledge, this is the first systematic study of bacterial gene expression in response to weak electric currents at the genome-wide scale. Since low-level electric currents can be delivered locally to medical devices and skin, the findings may be useful for developing more effective therapies.

## Materials and methods

### Bacterial strains and growth media

*B. subtilis *168 (*trpC2*) (Kunst et al. 1997) was used for planktonic studies. *B. subtilis *BE1500 (*trpC2*, *metB10*, *lys-3*, *ΔaprE66*, *Δnpr-82*, *ΔsacB::ermC*) (Jayaraman et al. 1999) was obtained from EI du Pont de Nemours Inc (Wilmington, DE) and used for the biofilm studies. Overnight cultures were grown at 37°C with aeration via shaking on an orbital shaker (Fisher Scientific; Hampton, NH) at 200 rpm. Biofilms were developed on 304L stainless steel coupons (5.6 cm by 1.0 cm) in batch culture at 37°C in 100 mm petri dishes (Fisher Scientific; Hampton, NH) for 48 h. Luria-Bertani (LB) medium (Sambrook and Russell 2001) consisting of 10 g/L NaCl, 10 g/L tryptone, and 5 g/L yeast extract (all from Fisher Scientific; Hampton, NH) was used for both planktonic and biofilm cultures. LB agar plates were prepared by adding 15 g/L Bacto agar (Fisher Scientific) to LB medium prior to autoclaving.

Poly-γ-glutamic acid (PGA) is a protein produced predominantly by members of the taxonomic order Bacillales (Candela and Fouet 2006) and is required for *B. subtilis *biofilm formation (Stanley et al. 2003). However, *B. subtilis *168 does not produce PGA, due to mutations in the *degQ *promoter region and the gene *swrA *(Stanley et al. 2003). Thus, *B. subtilis *BE1500, a strain which produces PGA and forms relatively good biofilms, was used for the study of *B. subtilis *biofilms.

### Electrochemical Cell Construction

**Figure 1:**
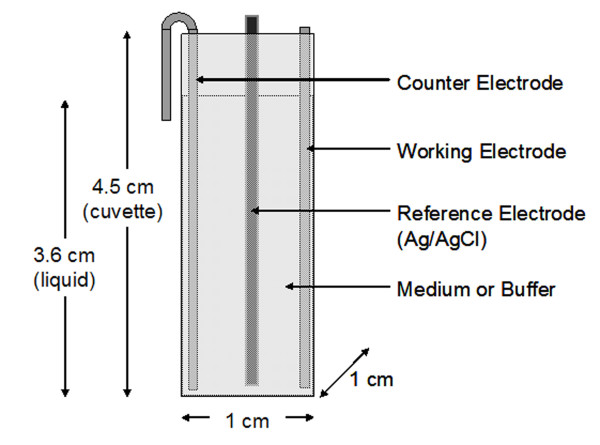
**Schematic of the electrochemical cell used in this study**. The reference electrode is Ag/AgCl wire inserted in a thin glass tube to prevent contact with the working or counter electrode. Biofilms grown on flat steel or carbon electrodes can be clipped onto the side; the liquid level is about 1 cm below the top of the cuvette when full (3 mL).

Electrodes with a dimension of 1 cm × 5.6 cm were cut from a 30.5 cm by 30.5 cm flat 304L stainless steel sheet (<0.08% C, 17.5-20% Cr, 8-11% Ni, <2% Mn, <1% Si, <0.045% P, <0.03% S; MSC; Melville, NY). Counter electrodes were bent at the end to form a hook shape (Figure 1). A counter electrode and working electrode were placed into a 4.5 mL standard-style polystyrene cuvette (Fisher Scientific; Hampton, NH). A 0.015" diameter silver wire (A-M Systems; Sequim, WA) was placed in bleach for 30 min to generate an Ag/AgCl reference electrode. The bottom 1" of a borosilicate glass Pasteur pipette (Fisher Scientific) was cut and the reference wire was placed inside to prevent accidental contact with the working or counter electrode. A potentiostat/galvanostat (Model #AFCBP1, Pine Instrument Company, Grove City, PA) was connected via alligator clamps to the electrodes and used to control the voltage and current. The volume of medium in the fully-constructed electrochemical cell was 3 mL. A schematic of the system is shown in Figure 1.

### Determination of Minimum Inhibitory Concentration and Minimum Bactericidal Concentrations

To determine the minimum inhibitory concentrations (MICs) of ampicillin on planktonic cells, *B. subtilis *168 and *B. subtilis *BE1500 were cultured in LB medium overnight as described above. The overnight cultures were subcultured by a 1:1000 dilution in LB medium containing various concentrations of ampicillin with seven replicates in a 96-well plate and allowed to grow at 37°C with shaking at 200 rpm for 24 h. The OD_600 _was measured immediately after inoculations and at 24 h after inoculation with a microplate reader (Model EL808, BioTek Instruments, Winooski, VT). The MIC was determined as the lowest concentration of ampicillin that completely inhibited growth.

MIC is not a useful measurement of the response of biofilms to antibiotics because antibiotics added in the growth medium before inoculation could kill planktonic cells before they can form a biofilm. Therefore it is important to characterize the minimum bactericidal concentration (MBC) of ampicillin on established biofilms. *B. subtilis *BE1500 was cultured overnight as described above. Flat stainless steel electrodes were placed in a 100 mm petri dish with 20 mL LB medium, which was inoculated with 20 μL of an overnight culture. Biofilms were allowed to develop for 48 h at 37°C without shaking. The electrodes with biofilms were gently washed three times in 0.85% NaCl buffer and immersed in LB medium containing various concentrations of ampicillin for 15 min. Immediately after treatment, the electrodes with biofilms were placed in a 15 mL polystyrene test tube (Fisher Scientific) containing 4 mL 0.85% NaCl buffer and sonicated for 2 min using a model B200 ultrasonic cleaner (Fisher) to remove the biofilm cells from the surface. The stainless steel electrode was then removed and the tube was vortexed for 30 s to break up any remaining cell clusters. CFUs were counted after spreading the buffer with cells on LB agar plates and incubated overnight at 37°C. The sonication steps were found safe to *B. subtilis *cells based on a CFU test (data not shown).

### Treatment of Planktonic Cells with DCs

*B. subtilis *168 was cultured overnight as described above, subcultured by a 1:1000 dilution in LB medium and grown to OD_600 _of 0.8. Cells from 3 mL of sub-culture were pelleted at 16,100 × g for 2 min in a microcentrifuge (Model 5415R Eppendorf, Westbury, NY), and resuspended in 0.85% NaCl buffer. This process was repeated three times to wash the cells, which were then resuspended in 3 mL LB or 3 mL pre-treated LB medium (see below). Samples in LB medium were treated for 15 min with a total current of 0, 150, 500, or 1500 μA (corresponding to 0, 25, 83 and 250 μA/cm^2^, respectively) in the electrochemical cell described above. Pre-treated LB media were prepared by treating LB medium with the same current levels for 15 min in the electrochemical cell described above. Cells were incubated in the pre-treated LB medium for 15 min without current to evaluate the cellular response to the chemical species generated by the currents, serving as control samples. Immediately after treatment, cells were aliquoted into microcentrifuge tubes, pelleted for 1 min at 16,100 × g and 4°C, and the supernatant decanted. The cell pellets were frozen immediately in a dry ice-ethanol bath and then stored at -80°C till RNA isolation.

### RNA Extraction

RNA extraction was performed using the RNeasy Mini Kit (Qiagen, Valencia, CA) by following the manufacturer's protocol with slight modifications. Briefly, the homogenization was performed with a model 3110BX mini bead beater and 0.1 mm diameter Zirconia/Silica beads (both from Biospec Products, Bartlesville, OK) for 1 min. On-column DNA digestion was performed with 120 μL DNase I; and wash with RPE buffer was repeated three times rather than once as described in the manufacture's protocol. The isolated RNA was stored at -80°C until DNA microarray analysis.

### DNA Microarray Analysis

The total RNA samples were sent to the DNA Microarray Core Facilities at SUNY Upstate Medical University for hybridization to GeneChip *B. subtilis *Genome Arrays (Affymetrix; Santa Clara, CA). The hybridizations were performed by following the Prokaryotic Target Preparation protocol in the GeneChip Expression Analysis Technical Manual (Affymetrix). cDNA was hybridized on DNA microarrays at 45°C for 16 h in a Model 640 Hybridization Oven (Affymetrix). The hybridized arrays were then washed and stained using the FS450_0004 protocol on an Affymetrix Fluidics Station 450. Finally, the arrays were scanned with a Model 7G Plus GeneChip Scanner (Affymetrix). For each data set, genes with a p-value of less than 0.0025 or greater than 0.9975 were considered statistically significant based on Wilcoxon signed rank test and Tukey Byweight. A cutoff ratio of 2 was also applied to these selected genes to ensure the significance of the results. Two biological replicates were tested for each condition. Cluster analysis was performed with the TIGR MultiExperiment Viewer (MeV) software (J. Craig Venter Institute; Rockville, MD) using a k-means sorting with the default parameters. Two biological replicates were tested for each condition.

### Treatment of Biofilm Cultures with Ampicillin and DC

*B. subtilis *BE1500 biofilms were prepared as described for MBC experiments. Prior to treatment, biofilms were gently washed three times with 0.85% NaCl buffer. Each stainless steel coupon with biofilm was placed as the working electrode in the electrochemical cell cuvette shown in Figure 1. Prior to placing the electrode with biofilm in the cuvette, 3 mL LB medium was added to the cuvette to prevent the biofilm from drying out. Samples were treated for 15 min with 0, 25, 83 and 250 μA/cm^2 ^DC. Immediately after treatment, the biofilms were placed in a 15 mL polystyrene test tube containing 4 mL 0.85% NaCl buffer and sonicated for 2 min to remove the biofilm cells from the electrode. The stainless steel electrode was then removed and the tube containing the cells and buffer was vortexed for 30 s to break up any remaining cell clusters. Cell densities after different DC treatments were determined by plating the cultures on LB/agar plates and counting CFUs. The effect of current-generated ions was tested in the same way except that the cells were incubated in pre-treated LB in the absence of a current.

### Atomic Force Microscopy

*B. subtilis *168 planktonic cells were cultured and treated with DCs as described above. Immediately after pelleting, the cells were centrifuged at 16,100 × g for 2 min at 4°C and the supernatant was decanted. Cell pellets were re-suspended in de-ionized (DI) water and centrifuged at 16,100 × g for 2 min at 4°C to wash away ions. The washing was repeated twice, and the pellet was resuspended in DI water. To prepare the samples for AFM analysis, 2 μL of suspended cells was placed on a piece of No. 2 borosilicate cover glass (VWR, West Chester, PA) and placed in a vacuum dessicator (Fisher Scientific) to dry for 15 min. Samples were examined using the contact mode of an atomic force microscope (Veeco Instruments; Malvern, PA). Both height and displacement images were captured at field widths of 50, 25, 10 and 5 μm.

## Results

### Effects of DCs on planktonic cells

To determine the effect of electrochemical currents on planktonic cells, *B. subtilis *168 cultures were grown overnight and treated in the custom built electrochemical cell (Figure 1) with total currents of 0, 150, 500 or 1500 μA, corresponding to 0, 25, 83 and 250 μA/cm^2^, respectively. To make a distinction between the effect of electrochemical reaction products and the current on the planktonic cells, cells were also incubated for 15 min in LB medium pre-treated with the same current level and duration (pre-treated LB medium). The number of viable cells was determined by CFU counts as described in the Materials and Methods section.

**Figure 2:**
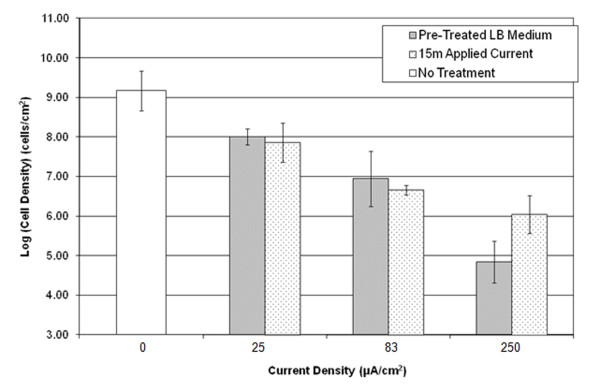
**Effects of DC and pre-treated media on planktonic cells of *B. subtilis *168**. Planktonic cells were sub-cultured to OD_600 _of 0.8, and 3 mL sub-culture was treated for 15 min at 37°C with no current, pre-treated medium, or applied current. CFUs were counted to determined cell viability after each treatment.

Planktonic cells exposed to pre-treated medium and applied current both showed a dose-dependent reduction of cell viability (Figure 2, one-way ANOVA, p < 0.0001). At 25 μA/cm^2 ^and 83 μA/cm^2^, both pre-treated LB medium and LB medium with applied current resulted in similar reduction of cell viability. For example, cell viability was reduced by approximately 1 log by 25 μA/cm^2^, and 2 logs by 83 μA/cm^2 ^vs. the untreated control. At 250 μA/cm^2 ^level, however, the pre-treated medium appeared to kill more cells than current treatment (4-log vs. 3-log reduction, two-way ANOVA nested model, p <0.0001).

### AFM analysis

**Figure 3:**
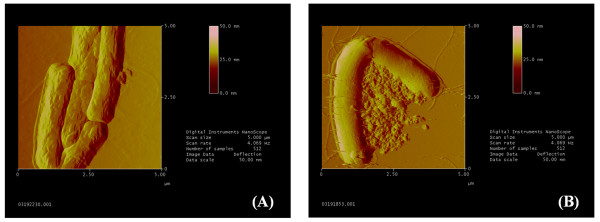
**Contact mode AFM images of cells treated with 500 μA total DC current (83 μA/cm^2^)**. Deflection mode images of planktonic *B. subtilis 168 *incubated with pre-treated LB medium at 25 μm (A), 5 μm (B) field size; or treated with 25 μA/cm^2 ^applied total current at 25 μm (C), 5 μm (D) field size.

To identify if DC treatments caused any physical damage to the cells, AFM analysis was performed to determine the effects of DCs on planktonic cell morphology. Cells were clearly visualized with high resolution using AFM (Figure 3). The images suggest that the width of the flagella to be less than 100 nm, the length to be at least 10 μm, and the wavelength to be approximately 2.5 μm. These numbers are in agreement with measurement of flagellar dimensions in the literature (Silverman and Simon 1977), suggesting that AFM is suitable for detecting detailed changes in cell morphology under our experimental condition. AFM images of *B. subtilis *168 in Figure 3 showed no apparent membrane features, appearing to be relatively smooth, consistent with an earlier report of AFM study that the membrane surface of *B. subtilis *W23 was observed to be smooth (Umeda et al. 1998).

As shown in Figure 3, treatments with DC did not cause apparent changes in cell morphology. Interestingly, during AFM and light microscopy, debris of an unknown type was observed, particularly in samples treated with 83 and 250 μA/cm^2 ^currents (Figure 3). To determine if this debris originated from the cells or from electrochemical reactions, LB medium without cells was treated with the same currents, washed, and analyzed in the same procedure. AFM images were taken at several resolutions (images not shown). There was an apparent increase in debris as the level of applied current increased. This debris was similar to the debris observed for samples containing cells in Figure 3. The apparent increase in debris with current suggests that these precipitates may be electrochemical reaction products and the results of their interactions with the components of LB medium. The AFM results suggest that the killing of bacterial cells by DC is not through direct physical forces of the currents (no change in the integrity of cells), but the electrochemical factors may play important roles. The effects of such debris on bacterial cells, however, remain to be determined.

### DNA microarray analysis

To understand the effect of electrochemical currents on *B. subtilis *at the genetic level, total RNA from planktonic *B. subtilis *168 treated with applied currents or pre-treated LB media were analyzed using GeneChip *B. subtilis *Genome Arrays (Affymetrix). *B. subtilis *168 cells treated with pre-treated LB media were used as controls to minimize the influence of electrochemical products on gene expression. In addition to grouping genes induced and repressed under each condition, cluster analysis was also performed to identify the genes induced only at one current level, up-regulation at all current levels, and down-regulation at all current levels.

As expected, the number of up-regulated genes increased with the current level. Treatment at 25, 83 and 250 μA/cm^2 ^DC significantly induced 12, 93 and 174 genes more than 2 fold, respectively. In comparison, the same treatments significantly repressed 11, 51 and 59 genes more than 2 fold, respectively. Consistent with the result that both pre-treated LB medium and LB medium with applied current caused similar reduction of cell viability (Figure 2), the genes under negative stringent control were not significantly repressed. This finding confirmed that the microarray data are useful for understanding the effects of current and ion movement. It is interesting to notice that although the number of induced/repressed genes increased with current level, the sets of genes changed are not inclusive. For example, among the 174 genes included by and 250 μA/cm^2 ^DC, 155 genes were induced only at this current level. Only genes *pstS *(expression ratio 2.5-7.7) and *yusU *(expression ratio 2.6) were induced at all current levels; and *srfAA *was repressed at all DC levels (2-4 fold). Despite the small number of genes induced/repressed at all conditions, there were 34 genes that were up-regulated (significantly changed based on p value but did not meet the two-fold ratio to be listed as "induced") at all tested current levels, and 4 that were down-regulated at all tested currents. A selected list of the genes can be seen in Tables 1, 2, 3, 4, 5 and 6. Full lists of differentially expressed genes can be found in the Additional File 1 (Supplemental Data).

**Table 1:** Genes induced by treatment with 25 μA/cm^2 ^DC.

Gene Name	Expression Ratio	Gene Function/product
*hisB*	2.1	imidazoleglycerol-phosphate dehydratase
*hisD*	2.1	histidinol dehydrogenase
*hisH*	2.1	imidazole glycerol phosphate synthase subunit HisH
*ilvH*	2.1	acetolactate synthase 3 regulatory subunit
*narJ*	2.0	nitrate reductase protein J
*narK*	2.1	nitrite extrusion permease
*pstS*	2.5	phosphate ABC transporter (binding lipoprotein)
*tuaA*	2.6	hypothetical protein
*tuaB*	2.2	colanic acid exporter
*tuaC*	2.1	glycosyltransferase
*ysnF*	2.0	*sigB *phosphate starvation induced protein
*yusU*	2.6	hypothetical protein

The genes are listed in the order of gene names. The experiment was done in duplicate and the average expression ratios of the two runs are shown.

**Table 2:** Genes repressed by treatment with 25 μA/cm^2 ^DC.

Gene Name	Expression Ratio	Gene Function/product
*arsB*	-2.3	arsenite efflux transporter
*arsC*	-2.1	arsenate reductase
*arsR*	-2.7	ArsR family transcriptional regulator
*cysC*	-2.1	adenylylsulfate kinase
*glmS*	-2.2	glucosamine--fructose-6-phosphate aminotransferase
*ileS*	-2.1	isoleucyl-tRNA synthetase
*iolB*	-2.2	5-deoxy-D-glucuronic acid isomerase
*mccB*	-2.5	cystathionine beta-lyase
*sat*	-2.3	sulfate adenylyltransferase
*srfAA*	-2.3	surfactin synthetase
*yqcK*	-2.6	putative thiol lyase

The genes are listed in the order of gene names. The experiment was done in duplicate and the average expression ratios of the two runs are shown.

**Table 3:** The 10 most induced genes by treatment with 83 μA/cm^2 ^DC.

Gene Name	Expression Ratio	Gene Function/product
*katA*	3.7	vegetative catalase 1
*pstA*	3.4	phosphate ABC transporter permease
*pstBA*	5.1	phosphate ABC transporter ATP-binding protein
*pstBB*	4.0	phosphate ABC transporter ATP-binding protein
*pstC*	3.5	phosphate ABC transporter permease
*pstS*	5.1	phosphate ABC transporter (binding lipoprotein)
*yxeK*	3.0	putative monooxygenase
*yxeL*	4.9	putative acetyltransferase
*yxeM*	3.2	putative ABC transporter binding lipoprotein
*yxeN*	3.0	putative ABC transporter permease

The genes are listed in the order of gene names. A full list of induced genes can be found in the Additional File 1 (Supplemental Data). The experiment was done in duplicate and the average expression ratios of the two runs are shown.

**Table 4:** The 10 most repressed genes by treatment with 83 μA/cm^2 ^DC.

Gene Name	Expression Ratio	Gene Function/product
*acoB*	-3.1	acetoin dehydrogenase E1 component (TPP-dependent beta subunit)
*gapB*	-3.4	glyceraldehyde-3-phosphate dehydrogenase
*mtlR*	-3.1	mannitol operon transcriptional regulator
*pyrP*	-3.0	uracil permease
*rbsD*	-4.9	D-ribose pyranase
*rbsK*	-3.2	ribokinase
*rbsR*	-3.2	LacI family transcriptional regulator
*xsa*	-4.0	alpha-L-arabinofuranosidase
*yhjR*	-5.9	putative electron carrier protein
*yncC*	-5.3	putative sugar transporter

The genes are listed in the order of gene names. A full list of repressed genes can be found in the Additional File 1 (Supplemental Data). The experiment was done in duplicate and the average expression ratios of the two runs are shown.

**Table 5:** The 10 most induced genes by treatment with 250 μA/cm^2 ^DC.

Gene Name	Expression Ratio	Gene Function/product
*narG*	6.7	nitrate reductase alpha subunit
*narJ*	4.8	nitrate reductase protein J
*pstC*	4.4	phosphate ABC transporter permease
*pstS*	7.7	phosphate ABC transporter (binding lipoprotein)
*tagG*	4.3	teichoic acid precursors permease
*tuaA*	5.5	hypothetical protein
*ygxB*	8.0	hypothetical integral membrane protein
*yhgD*	4.6	hypothetical transcriptional regulator
*yjgC*	4.1	putative oxidoreductase
*ypfB*	5.7	hypothetical protein

The genes are listed in the order of gene names. A full list of induced genes can be found in the Additional File 1 (Supplemental Data). The experiment was done in duplicate and the average expression ratios of the two runs are shown.

**Table 6:** The 10 most repressed genes by treatment with 250 μA/cm^2 ^DC.

Gene Name	Expression Ratio	Gene Function/product
*blyA*	-7.5	amidase
*glmS*	-4.8	glucosamine--fructose-6-phosphate aminotransferase
*ileS*	-3.0	isoleucyl-tRNA synthetase
*iolB*	-6.7	5-deoxy-D-glucuronic acid isomerase
*iolT*	-3.7	myo-inositol transporter
*mmsA*	-5.1	methylmalonate-semialdehyde dehydrogenase
*rbsD*	-3.6	D-ribose pyranase
*srfAA*	-4.0	surfactin synthetase
*valS*	-3.4	valyl-tRNA synthetase
*yomE*	-8.3	glycosyl hydrolase; phage SPbeta
		bacteriophage SPbeta N-acetylmuramoyl-L-alanine

The genes are listed in the order of gene names. A full list of repressed genes can be found in the Additional File 1 (Supplemental Data). The experiment was done in duplicate and the average expression ratios of the two runs are shown.

Sixteen genes were induced at both 83 and 250 μA/cm^2^. These genes include the *pst *operon (*pstS*, *pstC*, *pstA*, *pstBA*, *pstBB)*, a gene required for cytochrome bd production (*cydA*), and several genes encoding hypothetical proteins (*yddT*, *ygxB*, *yrhE*, *yusU*, and *ywtG*). In contrast, only five genes were induced at both 25 and 83 μA/cm^2 ^including three genes involved in histidine metabolism (*hisBDH*) and two encoding hypothetical proteins, e.g. *yusU*, and *pstS*. Interestingly, aside from *pstS *and *yusU*, five genes were induced at both 25 and 250 μA/cm^2^, but not at 83 μA/cm^2^. Most notable of these are *tuaABC*, responsible for teichuronic acid synthesis (Lahooti 1999; Soldo 1999); and *ysnF*, known to be induced during phosphate starvation (Antelmann 2000). All of these genes were also up-regulated to some degree below two-fold at 83 μA/cm^2^,

*B. subtilis *responds to stressors causing phosphate starvation by activating the *pho *regulon (Allenby 2004). The *pst *operon encodes proteins responsible for high-affinity phosphate uptake in conditions with low inorganic phosphate concentrations (Qi 1997). Genes in the *pst *operon (*pstS, pstA, pstBA, pstBB, pstC*) were found to be up-regulated at all tested currents based on the cluster analysis. *pstS *encodes a substrate-binding lipoprotein that is required for phosphate intake (Allenby 2004). This suggests that phosphate starvation may have occurred due to DC treatments.

At 250 μA/cm^2 ^level, 174 genes were induced. These genes include several encoding flagellar proteins (*flgBCM*), autolysins (*lytE*), sporulation regulators (*bofC, scoC, yaaH*), and competence delocalization (*mcsB*). Stress response genes up-regulated include heat shock genes *htpX *and *yflT*, general response genes *gspA *and *yfkM*, σ^G^-induced phosphate starvation gene *ysnF*, and an *yhdN *encoding NADPH specific aldo/keto-reductase. Additionally, five operons with unknown function were induced including *ydaDEGPS*, *yfhFLMP*, *yfkDJM*, *yjgBCD*, *and yxiBCS*..

At 83 μA/cm^2 ^several genes for ameliorating oxidative stresses were up-regulated, including those for uroporphyrinogen III synthesis (*hemBCDLX*), catalase, and a metalloregulated oxidative stress gene (*mrgA*). The genes for arsenic/antimony resistance (*arsBCR*, *yqcK*) were also up-regulated (although less than two fold).

### Effects of DC treatments on biofilms

**Figure 4:**
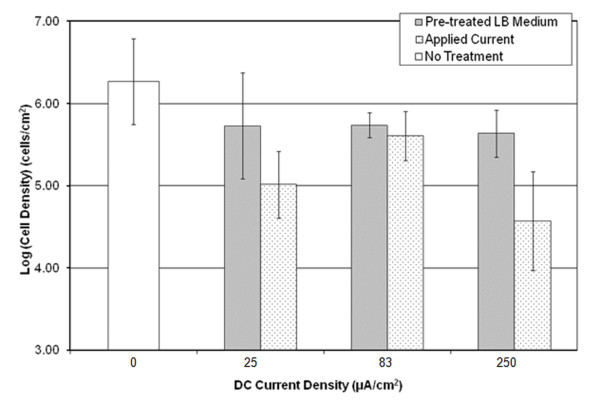
**Effects of DC and pre-treated media on biofilms of *B. subtilis *1500**. Biofilms grown for 2 days on 304L stainless steel electrodes at 37°C were treated with pre-treated LB medium or total applied current for 15 min as indicated. Cell density of the biofilms was calculated from the CFU data.

To determine the effect of DCs on biofilms, *B. subtilis *biofilms were developed on 304L stainless steel electrodes and treated with the same total DC levels as described for the planktonic cells (0, 25, 83, and 250 μA/cm^2^). To determine the effects of electrochemical reaction products on biofilms, biofilms were also treated with pre-treated LB media as with the planktonic cells. Immediately after treatment the biofilm cells were detached via sonication, washed with 0.85% NaCl buffer, and plated on LB-agar plates to quantify the number of viable cells by counting CFUs. A decrease in viability was seen for biofilm cells treated with all current levels as well as those treated with pre-treated LB media (Figure 4, one-way ANOVA, p < 0.01). Treatment with DC was more effective than pre-treated LB media at 25 and 250 μA/cm^2 ^(two-way ANOVA nested model, p < 0.05); while similar killing effects were observed at 83 μA/cm^2 ^(p = 0.98). CFU data showed that DC treatments at 25, 83 and 250 μA/cm^2 ^reduced cell viability by 97%, 88% and 98.5%, respectively.

**Figure 5:**
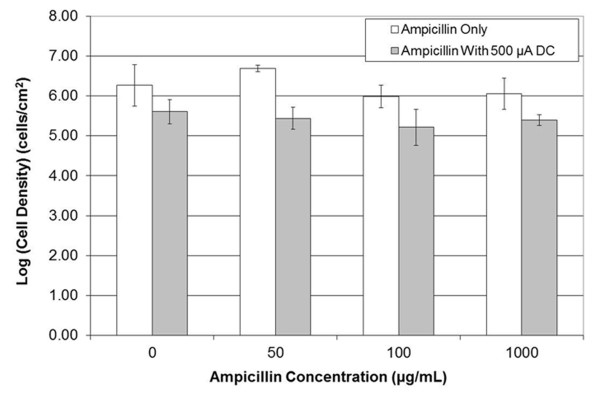
**Effects of ampicillin on biofilms of *B. subtilis *1500**. Biofilms were treated with varying concentrations of ampicillin and 500 μA total DC (83 μA/cm^2^) concurrently for 15 min at 37°C.

Consistent with the general knowledge that biofilms are highly tolerant to antibiotics, treatment of *B. subtilis *BE1500 biofilms with 1000 μg/mL ampicillin for 15 min only killed 59% of biofilm cells; while the MIC for planktonic *B. subtilis *BE1500 was found to be ≤ 2 μg/mL (data not shown), comparable to the MIC for *B. subtilis *168 of 0.2 μg/mL reported in the literature (Paudel et al. 2008). To determine if DCs can improve the control of *B. subtilis *biofilms with antibiotics, biofilms grown on 304L stainless steel electrodes were treated simultaneously with 0, 50, 100, or 1000 μg/mL ampicillin and 83 μA/cm^2 ^DC current for 15 min at 37°C. As discussed above, treatment with 83 μA/cm^2 ^DC current for 15 min alone decreased cell viability by 88%. In comparison, treatment with 50, 100 or 1000 μg/mL ampicillin in the presence of 83 μA/cm^2 ^DC decreased cell viability by 81%, 87%, and 89% versus antibiotic alone, respectively (Figure 5). Thus, no apparent synergy was found when treated with 83 μA/cm^2 ^DC and ampicillin together.

**Figure 6:**
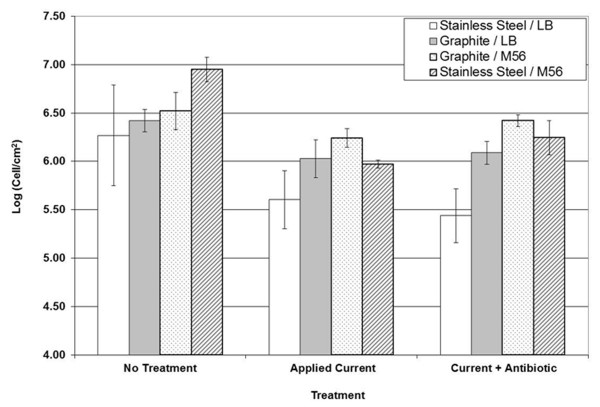
**Effects of electrode material and medium composition on the biofilm cells under DC treatment**. Biofilms were grown on graphite electrodes and treated with 500 μA DC current with and without 50 μg/mL ampicillin for 15 min at 37°C as indicated. Modified M56 buffer without chlorine was also tested as the electrolyte solution instead of NaCl buffer or LB medium.

Complex electrochemical reactions occur at the surface of electrodes when an external voltage is applied. The electrochemical generation of chlorine-containing species such as hypochlorite (ClO^-^), chlorite (ClO_2_^-^), and chloramines (NH_2_Cl, NHCl_2_, NCl_3_) by DC in the medium has been implicated in the killing of biofilm cells (Shirtliff et al. 2005). To understand if killing was partially due to hypochlorite generated by DC current, biofilms grown on graphite electrodes were also treated with chlorine-free M56 buffer. The viability of biofilm cells (with untreated control normalized as 100%) in M56 was 50% when treated with 83 μA/cm^2 ^DC alone, and 74% when treated with 83 μA/cm^2 ^DC current with 50 μg/mL ampicillin. Biofilms grown on stainless steel and treated with current with or without ampicillin in chlorine-free M56 buffer did not show significant difference in cell viability compared to those grown on stainless steel and treated in LB medium (Figure 6). This finding implies that the majority of killing of biofilm cells on stainless steel surfaces in LB medium was through the activity of metal ions, and may only minimally through chloride ions.

Ionic species can be generated from the electrode, and these may interact with the medium, antibiotics, and bacterial cells. The grade of stainless steel (304L) used in this study contains <0.08% C, 17.5-20% Cr, 8-11% Ni, <2% Mn, <1% Si, <0.045% P, and <0.03% S. Ions and compounds of some of these components could be toxic. For example Cr(VI), found in chromate and dichromate ions, is highly toxic to cells (Garbisu et al. 1998). To determine the effects of metal ions generated during treatment, biofilms were also grown on graphite electrodes rather than stainless steel (Figure 6). Treatment with 83 μA/cm^2 ^DC for 15 min reduced biofilm cell viability by 57% on graphite electrodes versus 88% on stainless steel. Treatment with 83 μA/cm^2 ^DC and 50 μg/mL ampicillin decreased cell viability by 44% on graphite electrodes versus 87% on stainless steel. Increases in viability of biofilm cells grown and treated on graphite electrodes compared to that on stainless steel suggest that metal ions released from the latter have stronger bactericidal effects on *B. subtilis *biofilms.

## Discussion

Here we report that treatment with low level DCs can effectively reduce the viability of *B. subtilis *cells. The effects of DCs and pre-treated media on the viability, morphology and gene expression of *B. subtilis *were studied. There was less killing of biofilm cells by incubating in the pre-treated media than when the current was directly applied, especially for biofilms treated with 250 μA/cm^2 ^(Figure 4). This finding suggests that the movement of ions or some transient species might be important for the killing of biofilm cells.

In contrast to the biofilm samples, planktonic cells were much more susceptible to DCs. However, planktonic cells exposed to current and to pre-treated media showed similar reduction in cell viability. It is possible that the presence of the biofilm matrix could reduce the effects of current-generated ions. The majority of the planktonic cells are not likely to be in direct contact with the electrode surface, especially given the vertical positioning of the electrodes (the turbidity in the cuvette appeared to be homogeneous). In contrast, biofilms are formed on the surface of the electrodes, positioned vertically, and held there by EPS. When a current is applied directly, biofilm cells are in direct contact with the metal cations released, possibly for the entire period of treatment as the ions were generated from the working electrode and diffused through the biofilm matrix. In the pre-treated LB medium, metal cations may have been converted to more inert forms relatively rapidly through reactions with water, oxygen, or hydroxide. In addition, biofilms treated with pre-treated LB media were not exposed to current directly; this may lead to a decreased exposure to metal cations, which were released from the anodic electrode. This can probably explain why treatments of biofilms with applied currents were more effective than using the pre-treated media prepared with the same level and duration of DC, especially at 250 μA/cm^2^. Precipitation of metal complex may also explain the additional killing by treating planktonic cells with 25 and 83 μA/cm^2 ^DC compared to pre-treated media. At 250 μA/cm^2^, however, applied DC was less effective than pre-treated media. This is probably due to the changes in electrochemistry, which may generate metal complex that are more effective than ions moving in an electric field as existed for treatments with DC. The exact nature of these reactions remains to be determined.

During electrochemical reactions involving stainless steel as the working electrode, a multitude of ions and other chemical species can be formed depending on the voltage and current levels and composition of the medium. In particular, the chemical species formed of five key elements are of particular interest with regards to cell viability include iron, chromium, chlorine, oxygen and hydrogen (pH). Fe^2+ ^ions can be generated during electrochemical reactions with stainless steel or graphite as an electrode (Dickinson and Lewandowski 1998). This effect may be intensified by the presence of biofilms on the stainless steel due to an increase in the resistance of the system, leading to an increased voltage when current is held constant (Dickinson and Lewandowski 1998). Ferrous ion can react with hydrogen peroxide via the Fenton reaction, resulting in the production of ferric ion, hydroxide ion, and the hydroxyl radical (Segura et al. 2008). This reaction has been reported to kill bacteria through further formation of the superoxide radicals (Andrews et al. 2003). In *B. subtilis*, oxidative stress due to H_2_O_2 _causes several genes to be up-regulated based on the response by the *per *regulon (Chen et al. 1995; Selinger et al. 2000). The induction of *katA *by 83 μA/cm^2 ^and of the *hemBCDLX *operon by 83 μA/cm^2 ^suggests that oxidative stress due to hydrogen peroxide may have been present. The decreased cell viability in biofilms treated with current may be in part due to oxidative stress as a result of the products of the Fenton reaction.

The second-most abundant metal in stainless steel is chromium, at amounts of up to 20% in 304L. Chromium ions, specifically Cr(VI) in chromate and dichromate, are highly toxic to bacterial cells (Garbisu et al. 1998). The presence and concentration of Cr(VI) in our system during treatment is unknown. *B. subtilis *168 has a metabolic pathway by which it can reduce Cr(VI) to the much less toxic Cr(III) that functions when chromate ions are present in concentrations of up to 0.5 mM (Garbisu et al. 1998). However, genes for chromate reduction (*ywrAB, ycnD*) did not show significant changes in expression under our experimental conditions. It has been reported that the presence of heavy metals, such as zinc, cadmium, and copper, can inhibit chromate reduction by *B. subtilis *(Garbisu et al. 1997). Genes related to zinc, cadmium, and copper toxicity (*copAB*) were induced in the presence of 250 μA/cm^2 ^current in our study. This finding suggests that ions of some heavy metals may be present in our system when using stainless steel as electrodes. Chromium reduction can also occur by chemical processes in solution, and can be enhanced or inhibited by other chemical species in the medium. Most significantly, the presence of Fe^2+ ^enables the reduction of Cr(VI) to Cr(III), at a ratio of 3 Fe^2+ ^to 1 Cr^6+^, possibly forming Fe/Cr complexes (Buerge and Hug 1997). However, the presence of organic ligands can modify this reaction; ligands specific for Fe^2+ ^inhibit the reaction, while those for Fe^3+ ^enhance it (Buerge and Hug 1998). In summary, the interactions of chromium within the system are complex, and killing via hexavalent chromium cannot be ruled out. However, the significant killing of *B. subtilis *using graphite electrodes suggests that the Cr(VI) ions are not indispensible for the killing effects of DC.

If metal cations are responsible for a loss of cell viability, we would expect to see genes that are related to metal tolerance to be up-regulated. Indeed, nine metal resistance genes were induced or up-regulated such as *arsBCR*, *appBCF and zosA *at 83 μA/cm^2^, and *copAB *at 250 μA/cm^2^. The *arsBCR *operon is responsible for the transport of arsenate, arsenite, and antimonite (Sato and Kobayashi 1998). These molecules bear little resemblance to divalent iron or hexavalent chromium compounds. It is interesting to note that arsenic is in the same group as phosphorous. It is possible that up-regulation of this operon may be related to the phosphate starvation.

In the absence of metal ions in solution as charge carriers, chloride ions in solution can react with hydroxyl ions to form hypochlorite, which is well known to be toxic to cells (Shirtliff et al. 2005). However, the experiments with graphite electrodes in M56 medium that did not contain chlorine showed that the metal ions are likely to be the dominating factors responsible for killing *B. subtilis *under our experimental conditions.

The bioelectric effect reported previously (Costerton et al. 1994) suggests that electric currents have a synergistic effect with antibiotics to improve the overall efficacy of killing biofilm cells. Surprisingly, in this study we observed that when ampicillin was added to the solution with current, the amount of killing was not significantly altered versus treatment with current alone. In the case of biofilms grown on graphite electrodes and treated in chlorine-free M56 buffer with 50 μg/mL ampicillin and 83 μA/cm^2 ^current there was even a slight decrease in killing. It is well documented that iron can interfere with the action of antibiotics, including ampicillin (Ghauch et al. 2009), through a variety of mechanisms including chelation of ferric cations by antibiotics (Ghauch et al. 2009; Nanavaty et al. 1998). It is possible that the presence of iron and other metal cations is inhibiting ampicillin activity through chelation mechanisms under our experimental condition. Such interaction may be dependent on the nature of antibiotics since some other antibiotics do show synergy with electric currents in killing biofilm cells (Costerton et al. 1994). It is also important to note that in this study we employed a shorter treatment time (15 min) than that in the study by Costerton and co-workers (24 h, Costerton et al. 1994). To obtain a deeper insight into the mechanisms at the molecular level, it will also be important to follow the kinetics of viability and gene expression over time.

In summary, we conducted a detailed study of the effects of weak DC on viability, gene expression and morphology of *B. subtilis*. The data suggest that the ions and oxidative species generated by electrochemical reactions have significant influence on bacterial gene expression and viability. Further testing with additional conditions and different antibiotics as well as study with mutants of key genes will help unveil the mechanism of bioelectric effects.

## Competing interests

The authors declare that they have no competing interests.

## Supplementary Material

Additional file 1 **Supplemental data**.. This file includes the full lists of included and repressed genes. Click here for file
