# Supplementary material for: Differential Gene Expression to Investigate the Effects of Low-level Electrochemical Currents on Bacillus subtilis
Source: AMB Express. 2011 Nov 11;1:39. doi: 10.1186/2191-0855-1-39 (PMC3294250; doi:10.1186/2191-0855-1-39)
Supplement: Additional file 1 — Supplemental data.. This file includes the full lists of included and repressed genes. [file 2191-0855-1-39-S1.PDF]

Supplemental Data

For

Differential Gene Expression to Investigate the Effects of Low-level Electrochemical Currents on *Bacillus subtilis*

Robert Szkotak[1,2], Tagbo H R Niepa[1,2], Nikhil Jawrani[1,2], Jeremy L Gilbert[1,2], Marcus B Jones[3] and Dacheng Ren[1,2,4,5*]

[1]Department of Biomedical and Chemical Engineering, Syracuse University, Syracuse, NY 13244, USA

[2]Syracuse Biomaterials Institute, Syracuse University, Syracuse, NY 13244, USA

[3]J. Craig Venter Institute, Rockville, MD 20850, USA

[4]Department of Biology, Syracuse University, Syracuse, NY 13244, USA

[5]Department of Civil and Environmental Engineering, Syracuse University, Syracuse, NY 13244, USA

[*]Corresponding author:

Dacheng Ren: Phone 001-315-443-4409. Fax 001-315-443-9175. Email: dren@syr.edu

Table S1. Genes induced at 25 µA/cm^2.

**Gene Name**	**Expression Ratio**	**Gene Function/product**
hisB	2.1	imidazoleglycerol-phosphate dehydratase
hisD	2.1	histidinol dehydrogenase
hisH	2.1	imidazole glycerol phosphate synthase subunit HisH
ilvH	2.1	acetolactate synthase 3 regulatory subunit
narJ	2.0	nitrate reductase protein J
narK	2.1	nitrite extrusion permease
pstS	2.5	phosphate ABC transporter (binding lipoprotein)
tuaA	2.6	hypothetical protein
tuaB	2.2	colanic acid exporter
tuaC	2.1	glycosyltransferase
ysnF	2.0	*sigB* phosphate starvation induced protein
yusU	2.6	hypothetical protein

Table 2. Genes repressed at 25 μA/cm^2.

Gene Name	Expression Ratio		Gene Function/product
arsB	0.44	-2.3	arsenite efflux transporter
arsC	0.47	-2.1	arsenate reductase
arsR	0.37	-2.7	ArsR family transcriptional regulator
cysC	0.48	-2.1	adenylylsulfate kinase
glmS	0.45	-2.2	glucosamine--fructose-6-phosphate aminotransferase
ileS	0.48	-2.1	isoleucyl-tRNA synthetase
iolB	0.45	-2.2	5-deoxy-D-glucuronic acid isomerase
mccB	0.41	-2.5	cystathionine beta-lyase
sat	0.44	-2.3	sulfate adenylyltransferase
srfAA	0.44	-2.3	surfactin synthetase
yqcK	0.38	-2.6	putative thiol lyase

Table S3. Genes induced at 83 µA/cm^2.

Gene Name	Expression Ratio	Gene Function/product
ahpF	2.5	alkyl hydroperoxide reductase large subunit
appB	2.6	oligopeptide ABC transporter permease
appC	2.3	oligopeptide ABC transporter permease
appF	2.0	oligopeptide ABC transporter ATP-binding protein
bltR	2.0	multidrug efflux transcriptional regulator
cydA	2.1	cytochrome bd ubiquinol oxidase subunit I
hemB	2.4	delta-aminolevulinic acid dehydratase
hemC	2.8	porphobilinogen deaminase
hemD	2.7	uroporphyrinogen-III synthase
hemL	2.1	glutamate-1-semialdehyde aminotransferase
hemX	2.7	negative effector of the concentration of glutamyl-tRNA reductase HemA
hisA	2.5	1-(5-phosphoribosyl)-5-[(5-phosphoribosylamino)methylideneamino] imidazole-4-carboxamide isomerase
hisB	2.8	imidazoleglycerol-phosphate dehydratase
hisD	2.5	histidinol dehydrogenase
hisG	2.5	ATP phosphoribosyltransferase catalytic subunit
hisH	2.8	imidazole glycerol phosphate synthase subunit HisH
hisI	2.3	bifunctional phosphoribosyl-AMP cyclohydrolase/phosphoribosyl-ATP pyrophosphatase protein
katA	3.7	vegetative catalase 1
mrgA	2.6	metalloregulation DNA-binding stress protein
mtnA	2.2	methylthioribose-1-phosphate isomerase
mtnW	2.1	2,3-diketo-5-methylthiopentyl-1-phosphate enolase
mtnX	2.1	2-hydroxy-3-keto-5-methylthiopentenyl-1-phosphate phosphatase
narG	2.2	nitrate reductase alpha subunit
nhaK	2.1	Na+/H+ antiporter
opuCA	2.5	glycine betaine/carnitine/choline/choline sulfate ABC transporter ATP-binding protein
opuCB	2.5	glycine betaine/carnitine/choline/choline sulfate ABC transporter permease
opuCC	2.4	glycine betaine/carnitine/choline/choline sulfate ABC transporter osmoprotectant-binding lipoprotein
ppsB	2.0	plipastatin synthetase
pstA	3.4	phosphate ABC transporter permease
pstBA	5.1	phosphate ABC transporter ATP-binding protein
pstBB	4.0	phosphate ABC transporter ATP-binding protein
pstC	3.5	phosphate ABC transporter permease
pstS	5.1	phosphate ABC transporter (binding lipoprotein)
sdpB	2.1	exporter of killing factor SpbC

sirB	2.1	sirohydrochlorin ferrochelatase
trpD	2.2	anthranilate phosphoribosyltransferase
yaaT	2.1	hypothetical protein
ycgA	2.1	putative integral inner membrane protein
ydbM	2.0	putative acyl-CoA dehydrogenase
yddT	2.1	hypothetical protein
ydiM	2.1	hypothetical protein
yfjE	2.2	putative integral inner membrane protein
ygxB	2.3	hypothetical integral membrane protein
yjnA	2.0	putative integral inner membrane protein
yqeH	2.4	GTPase involved in ribosome 30S assembly
yrhD	2.6	hypothetical protein
yrhE	2.5	putative formate oxidoreductase
yrkB	2.2	hypothetical protein
yrkC	2.1	putative dioxygenase; cupin family
yubD	2.2	putative efflux transporter
yumB	2.1	putative NAD-disulfide oxidoreductase
yumC	2.4	ferredoxin-NADP+ reductase
yusU	2.6	hypothetical protein
yusV	2.1	iron(III)-siderophore transporter ATP binding component
yvzA	2.1	hypothetical protein
ywqG	2.0	hypothetical protein
ywqK	2.1	hypothetical protein
ywtG	2.1	putative carbohydrate transporter
yxeK	3.0	putative monooxygenase
yxeL	4.9	putative acetyltransferase
yxeM	3.2	putative ABC transporter binding lipoprotein
yxeN	3.0	putative ABC transporter permease
yxeO	2.5	putative ABC transporter ATP-binding protein
yxeP	2.5	putative amidohydrolase
yxeQ	2.8	putative catabolic enzyme
yxeR	2.7	putative ethanolamine transporter
yybF	2.3	hypothetical permease
yybJ	2.6	ATP-binding cassette protein
yybL	2.3	hypothetical integral membrane protein
yybM	2.2	hypothetical integral membrane protein
yybT	2.5	phosphodiesterase
yydJ	2.2	putative antibiotic ABC transport permease
zosA	2.2	Zn transporter

Table S4. Genes repressed at 83 μA/cm^2.

Gene Name	Expression Ratio	Gene Function/product
acoB	-3.1	acetoin dehydrogenase E1 component (TPP-dependent beta subunit)
bglP	-2.6	phosphotransferase system (PTS) beta-glucoside-specific enzyme IIBCA component
bpr	-2.0	bacillopeptidase F
estB	-2.2	secreted esterase / lipase
exoA	-2.1	apurinic/apyrimidinic endonuclease
flhO	-2.1	flagellar basal-body rod protein
gapB	-3.4	glyceraldehyde-3-phosphate dehydrogenase
glgB	-2.0	glycogen branching enzyme
glnR	-2.0	nitrogen metabolism transcriptional regulator
glpF	-2.1	glycerol permease
gutR	-2.1	glucitol operon regulator
licA	-2.2	phosphotransferase system (PTS) lichenan-specific enzyme IIA component
licC	-2.4	phosphotransferase system (PTS) lichenan-specific enzyme IIC component
mcpA	-2.5	methyl-accepting chemotaxis protein
mmgB	-2.2	3-hydroxybutyryl-CoA dehydrogenase
mntB	-2.0	manganese ABC transporter ATP-binding protein
mntC	-2.5	manganese ABC transporter permease
msmE	-2.5	multiple sugar-binding lipoprotein
msmR	-2.8	LacI family transcriptional regulator
mtlR	-3.1	mannitol operon transcriptional regulator
nicK	-2.5	putative replication protein; mobile element region
pyrP	-3.0	uracil permease
rbsD	-4.9	D-ribose pyranase
rbsK	-3.2	ribokinase
rbsR	-3.2	LacI family transcriptional regulator
spoIIIAC	-2.0	stage III sporulation protein
srfAA	-3.0	surfactin synthetase
xsa	-4.0	alpha-L-arabinofuranosidase
ybeF	-2.1	hypothetical protein
ycnI	-2.1	hypothetical protein
ydcQ	-2.7	putative DNA wielding protein; mobile element region
ydcT	-2.1	hypothetical protein
yddA	-2.1	hypothetical protein
yddI	-2.1	hypothetical protein
yeaB	-2.5	putative cation efflux transporter
yesM	-2.5	two-component sensor histidine kinase
yesP	-2.1	rhamnogalacturonan permease
yheC	-2.1	spore coat associated protein
yhjR	-5.9	putative electron carrier protein

yknT	-3.0	hypothetical protein
yncC	-5.3	putative sugar transporter
yokE	-2.1	hypothetical protein
yotH	-2.0	hypothetical protein
yotK	-2.1	hypothetical protein
yqaC	-2.3	hypothetical protein
yrbE	-2.6	putative oxidoreductase
yusN	-2.0	hypothetical protein
ywsA	-2.2	hypothetical protein
yxeD	-2.0	hypothetical protein
yxkH	-2.6	putative exported polysaccharide deacetylase, lipoprotein
yyaD	-2.7	hypothetical integral membrane protein

Table S5. Gene induced at 250 μA/cm^2.

Gene Name	Expression Ratio	Gene Function/product
appD	2.9	oligopeptide ABC transporter ATP-binding protein
bofC	2.7	bypass of forespore C, intercompartmental signaling factor
cggR	3.0	transcriptional regulator of GapA
chaA	3.0	putative H+/Ca2+ antiporter
copA	2.1	copper transporter ATPase
copB	2.4	copper(I)-transporting ATPase
csbX	2.9	putative permease
cueR	2.4	copper efflux transcriptional regulator
cydA	2.1	cytochrome bd ubiquinol oxidase subunit I
cydB	2.7	cytochrome bd ubiquinol oxidase subunit II
cydC	3.1	ABC membrane transporter ATP-binding protein
cydD	3.4	ABC membrane transporter ATP-binding protein
cypC	3.0	fatty acid beta-hydroxylating cytochrome P450
czcD	3.1	potassium/proton-divalent cation antiporter
divIVA	2.0	cell-division initiation protein
endB	2.4	endoribonuclease antitoxin
ezrA	2.1	septation ring formation regulator EzrA
flgB	2.7	flagellar basal body rod protein FlgB
flgC	2.4	flagellar basal body rod protein FlgC
flgM	2.5	*sigD* repressor of flagellar synthesis
flhO	2.2	flagellar basal-body rod protein
fliE	2.0	flagellar hook-basal body protein FliE
fmnP	2.3	FMN permease
fnr	2.2	FNR/CAP family transcriptional regulator
fosB	2.1	fosfomycin resistance protein FosB (metallothiol transferase)
fruA	2.6	phosphotransferase system (PTS) fructose-specific enzyme IIABC component
fruK	3.0	fructose-1-phosphate kinase
fruR	2.2	fructose operon transcriptional repressor
gltA	3.2	glutamate synthase large subunit
gmk	2.5	guanylate kinase
gspA	2.1	glycosyl transferase (general stress protein)
hom	3.1	homoserine dehydrogenase
hprT	2.8	hypoxanthine-guanine phosphoribosyltransferase
htpX	2.3	heat shock protein HtpX
ilvE	2.1	branched-chain amino acid aminotransferase
lctP	2.4	L-lactate permease
lytE	2.2	cell wall hydrolase
maeA	2.1	malate dehydrogenase
manP	2.7	phosphotransferase system (PTS) mannose-specific enzyme IIBCA component
mcsB	2.1	ATP:guanido phosphotransferase

mgtE	2.5	magnesium transporter
mtnW	2.1	2,3-diketo-5-methylthiopentyl-1-phosphate enolase
nap	2.6	carboxylesterase NP
narG	6.7	nitrate reductase alpha subunit
narJ	4.8	nitrate reductase protein J
ndoA	2.1	endoribonuclease toxin
nsrR	2.9	NO-dependent activator of the ResDE regulon
nusB	2.3	transcription antitermination protein NusB
opuAA	2.7	glycine betaine ABC transporter ATP-binding protein
opuE	2.5	proline transporter
pdhA	2.6	pyruvate dehydrogenase (E1 alpha subunit)
pssA	2.0	phosphatidylserine synthase
pstA	3.5	phosphate ABC transporter permease
pstBA	3.5	phosphate ABC transporter ATP-binding protein
pstBB	2.7	phosphate ABC transporter ATP-binding protein
pstC	4.4	phosphate ABC transporter permease
pstS	7.7	phosphate ABC transporter (binding lipoprotein)
purE	2.5	phosphoribosylaminoimidazole carboxylase I
purK	2.8	phosphoribosylaminoimidazole carboxylase ATPase subunit
purR	2.3	pur operon repressor
purU	2.0	formyltetrahydrofolate deformylase
rsbV	2.2	anti-anti-sigma B factor
rsiW	2.1	anti-sigma(W) factor
scoC	2.8	transcriptional regulator of extracellular protease production and sporulation
tagG	4.3	teichoic acid precursors permease
tatAC	2.2	twin-arginine pre-protein translocation pathway protein
thyA	2.5	thymidylate synthase
tilS	2.5	tRNA(Ile) lysidine synthetase
tuaA	5.5	hypothetical protein
tuaB	3.2	colanic acid exporter
tuaC	2.5	glycosyltransferase
tuaD	2.3	UDP-glucose 6-dehydrogenase
xerC	2.0	site-specific tyrosine recombinase for chromosome partitioning
yaaH	2.2	spore peptidoglycan hydrolase
yaaQ	2.3	hypothetical protein
ybfM	2.0	putative membrane phosphatase
ycbP	2.7	putative inner integral membrane protein
ycdF	2.6	putative dehydrogenase
ycdG	3.4	putative glycosidase
ydaD	2.5	putative short chain dehydrogenase
ydaE	2.2	hypothetical protein
ydaG	2.2	putative general stress protein
ydaP	2.3	putative pyruvate oxidase

ydaS	2.1	hypothetical protein
ydbD	3.1	putative manganese-containing catalase
yddT	2.9	hypothetical protein
ydeO	2.1	putative integral inner membrane protein
ydgG	2.3	putative MarR family transcriptional regulator
ydgH	3.1	putative drug exporter of the RND superfamily
ydhI	2.0	putative acetyltransferase
ydhK	3.0	hypothetical protein
ydiR	2.0	BsuM restriction component
ydjG	2.3	putative phage replication protein
ydjH	2.2	hypothetical protein
ydjP	2.3	putative peroxydase
yeaA	2.2	hypothetical protein
yeaB	2.4	putative cation efflux transporter
yfhF	2.1	putative nucleotide binding protein
yfhL	2.6	SdpC immunity factor
yfhM	2.3	putative hydrolase
yfhP	2.1	putative membrane hydrolase
yfkD	2.9	hypothetical protein
yfkJ	2.1	protein-tyrosine-phosphatase
yfkM	2.4	general stress protein 18
yflA	2.8	putative aminoacid transporter
yflT	2.1	heat stress induced protein
yfmB	2.3	hypothetical protein
ygxB	8.0	hypothetical integral membrane protein
yhbI	2.4	putative MarR family transcriptional regulator
yhbJ	2.6	putative integral inner membrane protein
yhcA	2.6	putative exporter
yhcB	2.0	putative oxidoreductase
yhcW	2.8	putative phosphoglycolate phosphatase
yhdF	2.4	putative NAD(P)-dependent dehydrogenase
yhdN	2.9	aldo/keto reductase specific for NADPH
yhdT	2.2	hypothetical protein
yhgD	4.6	hypothetical transcriptional regulator
yhgE	2.0	hypothetical methyl-accepting protein
yhxD	3.4	putative oxidoreductase
yjcN	2.0	hypothetical protein
yjgB	2.3	hypothetical protein
yjgC	4.1	putative oxidoreductase
yjgD	3.2	hypothetical protein
yjzD	2.0	hypothetical protein
ykaA	2.8	putative Pit accessory protein
yknW	2.1	putative permease
ykuU	2.1	putative 2-cys peroxiredoxin
ykwB	2.4	putative acetyltransferase
ykzF	2.0	hypothetical protein

ylaN	2.0	hypothetical protein
ylbC	2.4	hypothetical protein
ylbN	2.5	hypothetical protein
ylmD	2.0	hypothetical protein
ylmE	2.1	hypothetical protein
yloC	2.6	hypothetical protein
yocB	2.0	hypothetical protein
yojN	2.1	putative nitric-oxide reductase
yonS	3.7	glycosyl hydrolase lipoprotein; phage SPbeta
yozB	2.5	putative integral inner membrane protein
ypfB	5.7	hypothetical protein
yphE	2.0	hypothetical protein
ypjP	2.1	hypothetical protein
yqhB	2.1	putative membrane associated protein
yqhQ	2.0	hypothetical protein
yqxL	3.1	putative CorA-type Mg(2+) transporter
yrdP	2.4	putative oxidoreductase
yrhE	3.2	putative formate oxidoreductase
yrhP	2.2	putative efflux transporter
yrkA	2.8	putative membrane associated protein
yrkL	2.1	putative NAD(P)H oxidoreductase
ysnF	3.1	*sigB* phosphate starvation induced protein
ythP	2.1	putative ABC transporter ATP-binding protein
ythQ	2.4	putative ABC transporter permease
ytzE	2.2	putative DeoR family transcriptional regulator
yuaI	2.1	putative acetyl-transferase
yubA	2.2	putative integral inner membrane protein
yueI	2.0	hypothetical protein
yukD	2.2	putative bacteriocin
yulF	2.2	biofilm formation protein
yusU	2.6	hypothetical protein
yuzA	2.4	hypothetical protein
yvbG	2.2	putative integral inner membrane protein
yvdS	2.1	putative small multidrug resistance protein
yvyF	2.0	*sigD*-regulated regulator of flagella formation
yvyG	2.4	flagellar protein
ywcJ	2.3	formate/nitrite transporter
ywmE	2.1	hypothetical protein
ywtG	2.6	putative carbohydrate transporter
ywzB	2.0	hypothetical protein
yxiB	2.1	hypothetical protein
yxiC	2.2	hypothetical protein
yxiS	2.1	hypothetical protein
yxjI	2.8	hypothetical protein

Table S6. Genes repressed at 250 µA/cm^2.

Gene Name	Expression Ratio		Gene Function/product
alaS	0.44	-2.3	alanyl-tRNA synthetase
aspS	0.44	-2.3	aspartyl-tRNA synthetase
bglS	0.45	-2.2	endo-beta-1,3-1,4 glucanase
blyA	0.13	-7.5	bacteriophage SPbeta N-acetylmuramoyl-L-alanine amidase
citB	0.47	-2.1	aconitate hydratase
dctP	0.38	-2.6	C4-dicarboxylate transporter DctA
dhbB	0.44	-2.3	isochorismatase
dhbC	0.47	-2.1	isochorismate synthase
efeN	0.50	-2.0	iron-dependent peroxidase convert ferric iron into ferrous iron
fumC	0.47	-2.1	fumarate hydratase
gapB	0.44	-2.3	glyceraldehyde-3-phosphate dehydrogenase
gatB	0.47	-2.1	aspartyl/glutamyl-tRNA amidotransferase subunit B
gcvPA	0.34	-2.9	glycine dehydrogenase subunit 1
gcvPB	0.50	-2.0	glycine dehydrogenase subunit 2
glcF	0.44	-2.3	glycolate oxidase iron-sulfur subunit
glmS	0.21	-4.8	glucosamine--fructose-6-phosphate aminotransferase
glpF	0.42	-2.4	glycerol permease
htpG	0.42	-2.4	class III heat shock protein 90
ileS	0.33	-3.0	isoleucyl-tRNA synthetase
iolB	0.15	-6.7	5-deoxy-D-glucuronic acid isomerase
iolC	0.41	-2.5	2-deoxy-5-keto-D-gluconic acid kinase
iolR	0.48	-2.1	DeoR family transcriptional regulator
iolT	0.27	-3.7	myo-inositol transporter
mmsA	0.20	-5.1	methylmalonate-semialdehyde dehydrogenase
mntA	0.38	-2.6	manganese ABC transporter manganese binding lipoprotein
mntB	0.34	-2.9	manganese ABC transporter ATP-binding protein
mntC	0.45	-2.2	manganese ABC transporter permease
moaD	0.41	-2.5	molybdopterin synthase small subunit
mrgA	0.45	-2.2	metalloregulation DNA-binding stress protein
nupC	0.39	-2.5	pyrimidine-nucleoside Na+(H+) cotransporter
pckA	0.41	-2.5	phosphoenolpyruvate carboxykinase
pdp	0.37	-2.7	pyrimidine-nucleoside phosphorylase
pheT	0.41	-2.5	phenylalanyl-tRNA synthetase subunit beta
pycA	0.47	-2.1	pyruvate carboxylase
rbsA	0.42	-2.4	ribose ABC transporter ATP-binding protein
rbsD	0.28	-3.6	D-ribose pyranase
rbsK	0.39	-2.5	ribokinase
rbsR	0.33	-3.0	LacI family transcriptional regulator
speD	0.50	-2.0	S-adenosylmethionine decarboxylase proenzyme
srfAA	0.25	-4.0	surfactin synthetase

tasA	0.47	-2.1	major biofilm matrix component
thrS	0.38	-2.6	threonyl-tRNA synthetase
valS	0.30	-3.4	valyl-tRNA synthetase
wapA	0.45	-2.2	cell wall-associated protein precursor
wprA	0.48	-2.1	cell wall-associated protease
yddR	0.48	-2.1	putative metal-dependent hydrolase
ydfQ	0.45	-2.2	putative thioredoxin or thiol-disulfide isomerase
yerA	0.42	-2.4	putative amidohydrolase
yesL	0.48	-2.1	putative permease
yhbB	0.50	-2.0	hypothetical protein
yneN	0.42	-2.4	putative membrane-bound protein with a thioredoxin-like domain
yoaU	0.50	-2.0	putative LysR family transcriptional regulator
yodL	0.48	-2.1	hypothetical protein
yojA	0.45	-2.2	putative H+/anion permease
yomE	0.12	-8.3	glycosyl hydrolase; phage SPbeta
yomI	0.47	-2.1	SPbeta phage protein; lytic transglycosylase
yozD	0.50	-2.0	hypothetical protein
ypzC	0.41	-2.5	hypothetical protein
yrbE	0.50	-2.0	putative oxidoreductase

Table S7. Genes induced at 83 $\mu A/cm^2$ only.

Gene Name	Expression Ratio	Gene Function/product
ahpF	2.5	alkyl hydroperoxide reductase large subunit
appB	2.6	oligopeptide ABC transporter permease
appC	2.3	oligopeptide ABC transporter permease
appF	2.0	oligopeptide ABC transporter ATP-binding protein
bltR	2.0	multidrug efflux transcriptional regulator
hemB	2.4	delta-aminolevulinic acid dehydratase
hemC	2.8	porphobilinogen deaminase
hemD	2.7	uroporphyrinogen-III synthase
hemL	2.1	glutamate-1-semialdehyde aminotransferase
hemX	2.7	negative effector of the concentration of glutamyl-tRNA reductase HemA
hisA	2.5	1-(5-phosphoribosyl)-5-[(5-phosphoribosylamino)methylideneamino] imidazole-4-carboxamide isomerase
hisG	2.5	ATP phosphoribosyltransferase catalytic subunit
hisI	2.3	bifunctional phosphoribosyl-AMP cyclohydrolase/phosphoribosyl-ATP pyrophosphatase protein
katA	3.7	vegetative catalase 1
mrgA	2.6	metalloregulation DNA-binding stress protein
mtnA	2.2	methylthioribose-1-phosphate isomerase
mtnX	2.1	2-hydroxy-3-keto-5-methylthiopentenyl-1-phosphate phosphatase
nhaK	2.1	Na+/H+ antiporter
opuCA	2.5	glycine betaine/carnitine/choline/choline sulfate ABC transporter ATP-binding protein
opuCB	2.5	glycine betaine/carnitine/choline/choline sulfate ABC transporter permease
opuCC	2.4	glycine betaine/carnitine/choline/choline sulfate ABC transporter osmoprotectant-binding lipoprotein
ppsB	2.0	plipastatin synthetase
sdpB	2.1	exporter of killing factor SpbC
sirB	2.1	sirohydrochlorin ferrochelatase
trpD	2.2	anthranilate phosphoribosyltransferase
yaaT	2.1	hypothetical protein
ycgA	2.1	putative integral inner membrane protein
ydbM	2.0	putative acyl-CoA dehydrogenase
ydiM	2.1	hypothetical protein
yfjE	2.2	putative integral inner membrane protein
yqeH	2.4	GTPase involved in ribosome 30S assembly
yrhD	2.6	hypothetical protein
yrkB	2.2	hypothetical protein
yrkC	2.1	putative dioxygenase; cupin family

yubD	2.2	putative efflux transporter
yumC	2.4	ferredoxin-NADP+ reductase
yusV	2.1	iron(III)-siderophore transporter ATP binding component
yvzA	2.1	hypothetical protein
ywqG	2.0	hypothetical protein
ywqK	2.1	hypothetical protein
yxeK	3.0	putative monooxygenase
yxeL	4.9	putative acetyltransferase
yxeM	3.2	putative ABC transporter binding lipoprotein
yxeN	3.0	putative ABC transporter permease
yxeO	2.5	putative ABC transporter ATP-binding protein
yxeP	2.5	putative amidohydrolase
yxeQ	2.8	putative catabolic enzyme
yxeR	2.7	putative ethanolamine transporter
yybJ	2.6	ATP-binding cassette protein
yybL	2.3	hypothetical integral membrane protein
yybM	2.2	hypothetical integral membrane protein
yybT	2.5	phosphodiesterase
yydJ	2.2	putative antibiotic ABC transport permease
zosA	2.2	Zn transporter

Table S8. Genes induced at 250 μA/cm^2 only.

Gene Name	Expression Ratio	Gene Function/product
appD	2.9	oligopeptide ABC transporter ATP-binding protein
bofC	2.7	bypass of forespore C, intercompartmental signaling factor
cggR	3.0	transcriptional regulator of GapA
chaA	3.0	putative H+/Ca2+ antiporter
copA	2.1	copper transporter ATPase
copB	2.4	copper(I)-transporting ATPase
csbX	2.9	putative permease
cueR	2.4	copper efflux transcriptional regulator
cydB	2.7	cytochrome bd ubiquinol oxidase subunit II
cydC	3.1	ABC membrane transporter ATP-binding protein
cypC	3.0	fatty acid beta-hydroxylating cytochrome P450
czcD	3.1	potassium/proton-divalent cation antiporter
divIVA	2.0	cell-division initiation protein
endB	2.4	endoribonuclease antitoxin
ezrA	2.1	septation ring formation regulator EzrA
flgB	2.7	flagellar basal body rod protein FlgB
flgC	2.4	flagellar basal body rod protein FlgC
flgM	2.5	*sigD* repressor of flagellar synthesis
flhO	2.2	flagellar basal-body rod protein
fliE	2.0	flagellar hook-basal body protein FliE
fmnP	2.3	FMN permease
fnr	2.2	FNR/CAP family transcriptional regulator
fosB	2.1	fosfomycin resistance protein FosB (metallothiol transferase)
fruA	2.6	phosphotransferase system (PTS) fructose-specific enzyme IIABC component
fruK	3.0	fructose-1-phosphate kinase
fruR	2.2	fructose operon transcriptional repressor
gltA	3.2	glutamate synthase large subunit
gmk	2.5	guanylate kinase
gspA	2.1	glycosyl transferase (general stress protein)
hom	3.1	homoserine dehydrogenase
hprT	2.8	hypoxanthine-guanine phosphoribosyltransferase
htpX	2.3	heat shock protein HtpX
ilvE	2.1	branched-chain amino acid aminotransferase
lctP	2.4	L-lactate permease
lytE	2.2	cell wall hydrolase
maeA	2.1	malate dehydrogenase
manP	2.7	phosphotransferase system (PTS) mannose-specific enzyme IIBCA component
mcsB	2.1	ATP:guanido phosphotransferase
mgtE	2.5	magnesium transporter
nap	2.6	carboxylesterase NP

narG	6.7	nitrate reductase alpha subunit
ndoA	2.1	endoribonuclease toxin
nsrR	2.9	NO-dependent activator of the ResDE regulon
nusB	2.3	transcription antitermination protein NusB
opuAA	2.7	glycine betaine ABC transporter ATP-binding protein
opuE	2.5	proline transporter
pdhA	2.6	pyruvate dehydrogenase (E1 alpha subunit)
pssA	2.0	phosphatidylserine synthase
purE	2.5	phosphoribosylaminoimidazole carboxylase I
purK	2.8	phosphoribosylaminoimidazole carboxylase ATPase subunit
purR	2.3	*pur* operon repressor
purU	2.0	formyltetrahydrofolate deformylase
rsbV	2.2	anti-anti-sigma B factor
rsiW	2.1	anti-sigma(W) factor
scoC	2.8	transcriptional regulator of extracellular protease production and sporulation
tagG	4.3	teichoic acid precursors permease
tatAC	2.2	twin-arginine pre-protein translocation pathway protein
thyA	2.5	thymidylate synthase
tilS	2.5	tRNA(Ile) lysidine synthetase
tuaD	2.3	UDP-glucose 6-dehydrogenase
xerC	2.0	site-specific tyrosine recombinase for chromosome partitioning
yaaH	2.2	spore peptidoglycan hydrolase
yaaQ	2.3	hypothetical protein
ybfM	2.0	putative membrane phosphatase
ycbP	2.7	putative inner integral membrane protein
ycdF	2.6	putative dehydrogenase
ycdG	3.4	putative glycosidase
ydaD	2.5	putative short chain dehydrogenase
ydaE	2.2	hypothetical protein
ydaG	2.2	putative general stress protein
ydaP	2.3	putative pyruvate oxidase
ydaS	2.1	hypothetical protein
ydbD	3.1	putative manganese-containing catalase
ydeO	2.1	putative integral inner membrane protein
ydgG	2.3	putative MarR family transcriptional regulator
ydgH	3.1	putative drug exporter of the RND superfamily
ydhI	2.0	putative acetyltransferase
ydhK	3.0	hypothetical protein
ydiR	2.0	BsuM restriction component
ydjG	2.3	putative phage replication protein
ydjH	2.2	hypothetical protein
ydjP	2.3	putative peroxydase
yeaA	2.2	hypothetical protein

yeaB	2.4	putative cation efflux transporter
yfhF	2.1	putative nucleotide binding protein
yfhL	2.6	SdpC immunity factor
yfhM	2.3	putative hydrolase
yfhP	2.1	putative membrane hydrolase
yfkD	2.9	hypothetical protein
yfkJ	2.1	protein-tyrosine-phosphatase
yfkM	2.4	general stress protein 18
yflA	2.8	putative aminoacid transporter
yflT	2.1	heat stress induced protein
yfmB	2.3	hypothetical protein
yhbI	2.4	putative MarR family transcriptional regulator
yhbJ	2.6	putative integral inner membrane protein
yhcA	2.6	putative exporter
yhcB	2.0	putative oxidoreductase
yhcW	2.8	putative phosphoglycolate phosphatase
yhdF	2.4	putative NAD(P)-dependent dehydrogenase
yhdN	2.9	aldo/keto reductase specific for NADPH
yhdT	2.2	hypothetical protein
yhgD	4.6	hypothetical transcriptional regulator
yhgE	2.0	hypothetical methyl-accepting protein
yhxD	3.4	putative oxidoreductase
yjcN	2.0	hypothetical protein
yjgB	2.3	hypothetical protein
yjgC	4.1	putative oxidoreductase
yjgD	3.2	hypothetical protein
yjzD	2.0	hypothetical protein
ykaA	2.8	putative Pit accessory protein
yknW	2.1	putative permease
ykuU	2.1	putative 2-cys peroxiredoxin
ykwB	2.4	putative acetyltransferase
ykzF	2.0	hypothetical protein
ylaN	2.0	hypothetical protein
ylbC	2.4	hypothetical protein
ylbN	2.5	hypothetical protein
ylmD	2.0	hypothetical protein
ylmE	2.1	hypothetical protein
yloC	2.6	hypothetical protein
yocB	2.0	hypothetical protein
yojN	2.1	putative nitric-oxide reductase
yonS	3.7	glycosyl hydrolase lipoprotein; phage SPbeta
yozB	2.5	putative integral inner membrane protein
ypfB	5.7	hypothetical protein
yphE	2.0	hypothetical protein
ypjP	2.1	hypothetical protein
yqhB	2.1	putative membrane associated protein

yqhQ	2.0	hypothetical protein
yqxL	3.1	putative CorA-type Mg(2+) transporter
yrdP	2.4	putative oxidoreductase
yrhE	2.9	putative formate oxidoreductase
yrhP	2.2	putative efflux transporter
yrkA	2.8	putative membrane associated protein
yrkL	2.1	putative NAD(P)H oxidoreductase
ythP	2.1	putative ABC transporter ATP-binding protein
ythQ	2.4	putative ABC transporter permease
ytzE	2.2	putative DeoR family transcriptional regulator
yuaI	2.1	putative acetyl-transferase
yubA	2.2	putative integral inner membrane protein
yueI	2.0	hypothetical protein
yukD	2.2	putative bacteriocin
yulF	2.2	biofilm formation protein
yuzA	2.4	hypothetical protein
yvbG	2.2	putative integral inner membrane protein
yvdS	2.1	putative small multidrug resistance protein
yvyF	2.0	*sigD*-regulated regulator of flagella formation
yvyG	2.4	flagellar protein
ywmE	2.1	hypothetical protein
ywzB	2.0	hypothetical protein
yxiB	2.1	hypothetical protein
yxiC	2.2	hypothetical protein
yxiS	2.1	hypothetical protein
yxjI	2.8	hypothetical protein
